# Comparison of Emergency Preparedness Practices between Food Assistance Program Participants and Non-Participants in the United States

**DOI:** 10.3390/ijerph182412937

**Published:** 2021-12-08

**Authors:** Gina J. Fung, Laura K. Jefferies, Michelle A. Lloyd Call, Dennis L. Eggett, Rickelle Richards

**Affiliations:** 1 Department of Nutrition, Dietetics, and Food Science, Brigham Young University, Provo, UT 84602, USA; gina.fung@va.gov (G.J.F.); laura_jefferies@byu.edu (L.K.J.); foodmichelle@gmail.com (M.A.L.C.); 2 Department of Statistics, Brigham Young University, Provo, UT 84602, USA; theegg@byu.edu

**Keywords:** emergency preparedness, food storage, water, low-income, food assistance programs

## Abstract

Background: Previous research has suggested many households are meeting the Federal Emergency Management Agency’s 3-day emergency food and water storage recommendations. The impact of limited economic household resources on emergency preparedness practices related to food and water is uncertain. The purpose of this study was to compare emergency preparedness practices in households participating in United States’ food assistance programs with households not participating in these programs. Methods: A convenience sample of adults (*n* = 572) completed an online Qualtrics survey. Descriptive statistics, chi-square statistics, and independent *t*-tests were used to measure differences between households participating in food assistance programs vs. non-participating households. Results: Most households participating in food assistance programs felt prepared to provide household members with food and water during an emergency, which did not significantly differ from non-participating households. Households using food assistance programs had less accessible cash but had similar foods on-hand for an emergency compared to non-participating households. However, they more frequently reported having baby formula/food and less frequently reported having vitamin/mineral supplements compared to non-participating households. Conclusions: Food assistance programs may be effective in providing enough food and water to help low-income families be prepared for an emergency.

## 1. Introduction

The United States (US) experiences more than 100 natural disasters annually, affecting millions of people and costing an average of $121.3 billion in damages [1,2]. With 2020 being a record-breaking year for climate and weather-related events, it is projected that the number of some types of disasters will continue to increase [1,2]. The Federal Emergency Management Agency (FEMA), part of the Department of Homeland Security, is responsible for coordinating disaster relief and increasing emergency preparedness in the US. FEMA recommends that each household have a basic emergency supply kit that includes one gallon of water per person per day for at least three days, and at least a three-day supply of non-perishable food [3]. The 2017 American Housing Survey showed that 58.6% of Americans had at least three gallons of water stored for each person in the household and 81% of Americans had enough non-perishable food to last everyone in the household for three days [4]. According to the 2020 National Household Survey, 51% of the population considered themselves as prepared overall for an emergency, which was a mere increase of 2% since 2013 [5]. 

Golem and Byrd-Bredbenner [6] found that food storage among food secure households in New Jersey exceeded recommendations for longevity and nutritional adequacy. Likewise, Hiatt et al. [7] reported that while the vast majority of nationwide survey respondents (*n* = 572) have enough food on hand, only 53% have enough water to meet FEMA’s preparedness standard. While most US households appear to be prepared for emergencies, the same may not be true for low-income households.

The 2021 weekly Household Pulse Survey estimates that as many as 12.7% of Americans reported not having enough food within the past seven days [8]. The link between low economic status and disaster preparedness indicates that those with lower economic status are not as ready as others, because many of these preparations are beyond their fiscal means [9]. Of this group, individuals and families that participate in food assistance programs in the US have limited economic resources, and thus may be less prepared for an emergency. 

Food assistance programs in the US include federally-funded government programs that provide food or cash benefits to low-income households, such as the Supplemental Nutrition Assistance Program (SNAP), the Special Supplemental Nutrition Program for Women, Infants and Children (WIC), National School Lunch Program (NSLP), and School Breakfast Program (SBP), and initiatives run by non-profit organizations (e.g., food banks), religious organizations, or other entities that provide food to those in need. SNAP eligibility requires households with or without children to have annual household incomes 130% or below the US poverty guidelines and provides households with cash benefits to purchase allowable foods with an Electronic Benefits Transfer card [10]. WIC is a program for low-income (annual household income 185% or below the US poverty guidelines) pregnant, postpartum, and breastfeeding women, infants, and children up to 5 years of age and provides eligible individuals with nutrient-rich foods [11]. Low-income households with school-aged children can also qualify for free and reduced-price meals through the NSLP and SBP based on annual household income (free, 130% or below the US poverty guidelines and reduced, 130–185% of the US poverty guidelines) [12].

Food assistance programs aim to provide relief for food insecurity, but it is uncertain whether or not these programs help households be more prepared for an emergency. Whether those who rely on such programs also look to their local, state, and federal government to assist them during natural disasters is also unknown. Thus, the purpose of this study was to evaluate the food and water preparedness of households on food assistance programs compared to those that are not. We hypothesized that those participating in food assistance programs would be less prepared for an emergency, including food and water storage, compared to those not participating in food assistance programs.

## 2. Materials and Methods

### 2.1. Study Design & Sample 

A cross-sectional study design was used to collect data about food and water emergency preparedness perceptions and practices from adults residing in the US (*n* = 572). A convenience sample of Qualtrics (Qualtrics, Provo, UT, USA) survey panelists took a 142-item online survey in 2014. The survey was developed based on underlying emergency preparedness guidelines for the general public provided by FEMA, the Centers for Disease Control and Prevention’s Behavioral Risk Factor Surveillance Survey (BRFSS) [13]. Further details about survey development have been published elsewhere [7].

Panelists initially received an invitation to participate, and if interested, completed screening questions to determine eligibility. Respondents provided consent to participate in the study by clicking the “next” button to view the survey questions and agreed that completion of the survey would indicate their consent. Respondents were compensated for their time based on Qualtrics panel rates. The Brigham Young University’s Institutional Review Board approved this study.

Respondents who indicated use of WIC, SNAP, food banks, religious organization resources, reduced or free school meals for children in the household, and/or other food assistance programs were categorized as “Food Assistance Program Participants”. Respondents who indicated that they did not participate in any food assistance programs were categorized as “Non-participants”. In the present study, comparisons of emergency preparedness variables were made between Food Assistance Program Participants and Non-participants.

### 2.2. Emergency Preparedness Variables 

For the current paper, a subset of 31 survey items were used to describe respondents’ perceptions of general emergency preparedness and for providing food and water for a respondent’s household in an emergency; the number of different kinds of non-perishable foods available at home, categorized by USDA MyPlate food groups [14] (USDA, MyPlate https://www.myplate.gov/ (accessed on 21 October 2021); the availability of and types of water stored for an emergency; the length of time food, water, and accessible cash could sustain household members in an emergency; respondents’ perceived responsibility of household, local, state, and federal government in taking care of their household in an emergency; and demographic characteristics. Table 1 summarizes the survey questions by category, definitions provided to respondents, and survey response options. 

Food storage practices were measured through a series of questions that asked respondents to identify which frozen and non-perishable food items were available at home (Table 1). If respondents affirmed having non-perishable foods and/or frozen foods in their household, subsequent questions provided lists of foods based on MyPlate food groups [14] (USDA, MyPlate https://www.myplate.gov/ (accessed on 21 October 2021) and other similarities, as outlined in Table 1.

### 2.3. Statistical Analysis

Descriptive statistics, including means and frequencies, were used to describe the data. For each respondent, a sum total of items selected under each of the food lists was calculated, representing the number of different kinds of non-perishable foods available in the household based on MyPlate food groups, non-perishable non-dairy beverages, and other non-perishable foods. Means were then calculated across the sample, with comparisons between food assistance program participants and non-participants made. A sample size calculation indicated that a sample of *n* = 126 would be sufficient to detect a 0.36 mean (standard deviation = 1) difference (on a 5-point Likert scale) at 80% power and an alpha level of 0.05.

For the survey question about the length of time food could last the household, we merged the third and fourth response options into one category—“more than 3 days, but less than 1 month”—and we merged the last four options into “at least 1 month or more.” For the survey question about the length of time water could last the household, we merged third and fourth response options into one category—“more than 3 days, but less than 1 month”—and we merged the last two categories into “at least 1 month or more.” For the accessible cash survey question, we combined the last three categories into one—“at least 6 months or more”.

Respondents’ US state of residence was classified into a geographical region (Midwest, Northeast, South, and West) based on the US Census Bureau classifications [15]. Respondents’ zip codes were classified into Rural-Urban Commuting Area Codes (RUCA) as established by the US Department of Agriculture [16]. One respondent’s data showed inconsistencies between zip code, county, and state, and thus was excluded in RUCA analyses.

Independent *t*-tests and chi-square statistics were used to evaluate differences between food assistance program participants and non-participants. A significance level of *p* < 0.001 was used to account for multiple comparisons bias. All analyses were performed in Statistical Analysis System (SAS) software, version 9.2 (SAS Institute Inc., Cary, NC, USA, 2007).

## 3. Results

A total of 1360 subjects clicked on the survey link, with 42% (*n* = 572) included in all statistical analyses. Data were excluded because of incomplete respondent data (*n* = 124 for incorrectly answering an attention filter; *n* = 648 for exiting the survey before completing it), and short survey completion rate (<10 min, *n* = 12). Four respondents had a duplicate IP address, so only their first response was used.

Table 2 provides demographic characteristics of the sample. Twenty-two percent of respondents were categorized as “Food Assistance Program Participants” (*n* = 126). Respondents who indicated that they did not participate in any food assistance programs were categorized as “Non-participants” (*n* = 446). Compared to non-participants, food assistance program participants were younger, had less education, were unemployed, had less time that accessible cash could provide for household needs in an emergency, perceived overall health rating as less favorable, and reported a higher mean number of children in the household.

The majority of respondents felt somewhat prepared for emergencies overall, including the ability to provide food and water for their households in an emergency, with no differences between food assistance program participants and non-participants in any of these areas (Table 3). Close to one-third of respondents in both groups reported owning a disaster supplies kit. Between the groups, there was also no difference in perceived government and individual responsibilities for emergency preparedness.

On average, the respondents from both groups had approximately 10 different kinds of grain-based foods, 1 kind of fruit, 3 kinds of vegetables, 4 kinds of protein foods, and 2 kinds of dairy foods (Table 4). However, no differences were evident between food assistance program participants and non-participants for any of the food groups. A higher percentage of food assistance program participants had baby formula and baby foods at home than non-participants. A lower percentage of food assistance program participants had vitamin and mineral supplements at home, compared to the non-participants. Over 90 percent of the respondents in both groups indicated that they had enough food to feed their household for more than 3 days. 

Overall, there were no differences in emergency water storage practices between food assistance program participants and non-participants (Table 5). About 63% of all the respondents have water storage available for emergencies. Among those who had water stored, 45% of food assistance program participants and 55% of non-participants could provide water for the household in an emergency for at least 3 days.

## 4. Discussion

To our knowledge, this is the first published study evaluating food and water storage practices among households using food assistance programs in the US. Overall, our results suggested that households participating in food assistance programs feel equally as prepared in providing household members with food and water during an emergency as those who do not rely on food assistance programs. More than 90% of households in the study who participated in food assistance programs reported having 3 or more days worth of food that could be used to meet their households’ typical eating patterns during an emergency, suggesting they were meeting FEMA guidelines for food storage at home [17]. However, about 55% of households participating in food assistance programs did not meet FEMA guidelines for having at least 3 days worth of water storage [17].

Our study found that most households using food assistance programs have enough food on-hand to sustain their household for at least 3 days after an emergency and that their perceived level of food storage availability in being prepared to provide their household with food during an emergency did not differ from households who were not participating in food assistance programs. Gladwin and Peacock [18] found that households with lower socioeconomic status were equally prepared with supplies for Hurricane Andrew as higher-income households. In contrast, Morrow and Enarson [19] found that women with low-income status had limited economic means to obtain emergency supplies to prepare for the impending Hurricane Andrew. From these previous studies, it is unknown whether the low-income households were participating in food assistance programs, what foods were on-hand already, and how long the food could last. Our study offers insight that households participating in food assistance programs may fare well after an emergency for at least 3 days if they can stay at their home base, especially in having different kinds of grains and cereal-based foods and protein foods available. However, if households had to leave their home-base they may be limited in the amount of food they could transport elsewhere, especially considering the number of food assistance program participants in our study (40%) who reported having less than one week’s worth of accessible cash to meet their household’s basic needs. Collectively, our study findings suggest food assistance programs may provide households with enough benefits to help most of them maintain an adequate emergency household food supply for at least 3 days.

The significant difference in household availability of baby formula, cereal, and foods between food assistance program participants and non-participants was not surprising given WIC is a food assistance program that provides these foods for households with infants [20]. On average, respondents in our study who participated in food assistance programs had significantly more children in the home than non-participants, thus likely resulting in participating in programs like WIC. Availability of WIC infant foods among our study participants may prove beneficial in the aftermath of a disaster if households still had access to their home food supply [19].

Food assistance program participants less frequently reported having vitamin/mineral supplements available than non-participants. Our findings support previous research that found lower multivitamin/mineral use among SNAP participants than those not eligible for or on SNAP [21]. Given food assistance programs do not provide nutrient supplements as part of their benefits, households using food assistance programs may have limited personal funds to spend on these items. Having access to multivitamin/mineral supplements may be important for some populations after an emergency, such as pregnant women [22] and older adults [23].

Perceptions of being prepared to provide water for the household in an emergency were similar across food program participants and non-participants in our study, with around 40% feeling unprepared. Further, no differences were evident between food assistance program participants and non-participants related to the length of time their stored water could sustain household members in an emergency. Only about half of the respondents in each group reported their stored water could last at least 3 days, which is similar to previous research among US households [24,25].

Unique to our study was the finding that food assistance program participants less frequently reported storing water in commercially-packaged containers compared to non-participants. Some food assistance programs like WIC do not provide bottled water as eligible food items, and while other programs like SNAP would allow participants to purchase these items, those in our study were less frequently doing so. It is possible that households placed purchasing these items as a lower priority because of access to a clean, safe water supply in the US and the more affordable option was to refill personal containers. Although using personal containers can be a viable option, FEMA now recommends storing commercially-packaged water for an emergency [3]. Thus, based on our study findings, households participating in food assistance programs may not be as able to meet this recommendation as those not participating in these programs.

Eligibility for food assistance programs requires households to have limited income, thus explaining the short sustainability of accessible cash among respondents in the current paper. A Hurricane Katrina study published in 2007 showed that a lack of accessible cash for gas and travel expenses made it difficult for low-income African American families to evacuate before the disaster [26]. Based on our findings, families that rely on food assistance programs may be discouraged from evacuating their homes when facing an emergency due to their inability to afford or access transportation or repurchase food and other supplies upon relocation.

FEMA recommends saving money in an emergency savings account and having cash available to make purchases in an emergency [27]. Almost one-third of respondents could not rely on accessible cash to provide basic household needs, including water and food, for up to 3 months. This may be explained by several factors. The 2016 Survey of Consumer Finances reported that 24 percent of households had less than $400 in liquid assets after covering monthly expenses [28]. The increased use of debit and credit cards and other electronic payment options over the past 20 years, along with occasional fees associated with ATM withdrawals and “cash back” transactions may also contribute to this [29]. Cash on-hand is also related to increased crime which may also deter some from having it readily accessible [30].

The low incidence of having an emergency supply kit in this study is consistent with reports of the low and varied prevalence of emergency supply kits across all US households [3]. While there are countless sources on what to include in a kit, the lack of consistent content recommendations, including those that are disaster type-specific, and possible lack of knowledge on their usefulness, appears to impact Americans equally regardless of the level of potential resources [31]. That there was no difference between those receiving and not receiving food assistance in our study suggests that reliance on the food assistance programs does not equate to an expectation that the government provides for their needs during a crisis any more than others. Similarly, Tyler and Sadiq [32] concluded that income level was unrelated to reliance on local, state, and federal government resources in an emergency.

### Limitations

The term “well-prepared” for a disaster was not defined in the survey, so respondents may have interpreted it differently. For the food variables, we simply asked whether or not respondents had each particular item available in their home, not how much they had of each. Also, we measured respondents’ perceptions about the length of time their food supply could last to feed their household in an emergency rather than measuring the actual amount of food available. Further, we were unable to document the proportion of the household’s food supply that came from a food assistance program vs. personal funds. Although income-eligibility criteria for food assistance programs indicate households in our study would be low-income, it is possible that low-income households were in the “non-participants” category in our study. Thus, inferences to our study can only be made for those participating in food assistance programs vs. those not participating. While this was a nationwide survey, the results are a convenience sample and may not be reflective of all geographic regions in the US. Race/ethnicity is not representative of the US population.

It is important to note that responses from participants and non-participants alike, may only reflect their food and water supply at the time the survey was taken. Quantities of food and food category can fluctuate throughout an income or assistance period or a shopping trip. Types of food on hand may vary by season or event. Previous research has shown that among those on food assistance programs or low-income households, fluctuations in household economic resources occur throughout the month, thus perceptions of how long supplies could last and actual kinds of foods may depend on time frame [33]. 

## 5. Conclusions

Food assistance programs may be effective in providing enough food, especially a variety of grain-based and protein-based foods, for participating households in an emergency. However, our study found that households participating in food assistance programs are equally unprepared to provide an adequate supply of water during an emergency as non-participating households, suggesting more efforts to improve preparedness are warranted for all US households. Strategies to improve water preparedness could include more effective outreach and promotion of existing US federal emergency preparedness initiatives, such as FEMA’s *Ready* campaign [34] or enhancing partnerships between individuals, communities, and government [35,36]. Establishing strong social networks among US citizens who promote emergency water storage may also help households be more prepared [37,38,39]. As limited-income status is a requirement to participate in food assistance programs, limited accessible cash and low presence of emergency kits among such participants, compared to non-participants, may present challenges in these households meeting relocation demands during an emergency due to a lack of resources. Future research should evaluate actual amounts of food and water stored for an emergency among households participating in food assistance programs and the proportion of a household’s food supply provided through food assistance programs versus personal funds.

## Figures and Tables

**Table 1:** Survey questions by category, associated definitions, and survey response options.

Survey Question by Category	Definitions and Response Options
General, food, and water emergency preparedness	
“Overall, how well prepared do you feel your household is to handle a large-scale disaster or emergency?” “How well prepared do you feel your household is to do each of the following actions in a large-scale disaster or emergency?” (Provide water for my household, provide food for my household)	*Definition*: “Any event that leaves you isolated in your home or displaces you from your home for at least 3 days. This might include natural disasters such as earthquakes, hurricanes, tornadoes, floods, ice storms, or man-made disasters such as explosions, terrorist events, or blackouts.” *Response Options*: Well prepared, somewhat prepared, not at all prepared
Frozen * and/or non-perishable foods	
“Which of the following foods do you have stored in your freezer(s)?” “The following is a list of non-perishable grain and cereal-based foods [fruits, protein foods, vegetables, milk products, drinks, items] that you may have in your home. Please mark any of the items that you currently have in your household. If you have any amount of the food, please mark it.”	*Definition*: “Foods that do not require refrigeration such as canned foods, staples (flour, sugar, grain, pasta, etc.), dry mixes (dry soup mixes, macaroni and cheese, cake mixes, etc.), dry ready-to-eat cereals, snack foods (crackers, chips, etc.), [and] food set aside for emergency situations.” *Response Options Grouped by MyPlate:* *Grain and cereal-based foods*: brown rice, white rice, wheat kernels/unground wheat,^†^ oats/oatmeal, ready-to-eat cereal, pasta, packaged rice or pasta seasoned meals/side dishes, cornmeal/masa flour, whole wheat flour,^†^ white flour,^†^ low-sodium crackers, regular crackers, pretzels, corn chips/tortilla chips, pancake/waffle mix, granola bars or fruit grain bars, popcorn, cake mixes, packaged cookies, and bread/rolls/bagels/tortillas. ^†^ *Fruits*: commercially canned/bottled fruits (not jams/jellies), home-canned/bottled fruits (not jams/jellies), commercially dried/dehydrated fruits (i.e., raisins, fruit leather, dried apricots), home-dried/dehydrated fruits (i.e., raisins, fruit leather, dried apricots), and frozen fruit. *Vegetables*: commercially canned/bottled vegetables, home-canned/bottled vegetables, commercially dried/dehydrated vegetables (i.e., dried carrots, tomatoes), home-dried/dehydrated vegetables (i.e., dried carrots, tomatoes), potato chips, instant mashed potatoes, and packaged potato meals/side-dishes (i.e., scalloped, hash browns, cheesy), and frozen vegetables. *Protein foods*: commercially canned/bottled meat (includes tuna or other fish, chicken/turkey, beef, pork, etc.), home-canned/bottled meat (includes poultry, beef, pork, fish, etc.), commercially dried/dehydrated meats (i.e., jerky, bacon bits, fish or seafood, meat substitutes), home-dried/dehydrated meats (i.e., jerky, bacon bits, fish or seafood, meat substitutes), frozen meats (includes poultry, beef, pork, fish, meat substitutes, etc.), commercially canned bottled beans/legumes (not green beans), home-canned bottled beans/legumes (not green beans), dry beans/legumes, frozen beans/legumes (not green beans), nuts, ^†^ sunflower seeds or pumpkin seeds, peanut butter, and trail mix. *Dairy*: powdered milk, powdered milk alternatives (i.e., soy, almond, rice), hot cocoa mix, boxed (shelf-stable) fluid cow’s milk, boxed (shelf-stable) fluid milk alternatives (i.e., soy, almond, rice), canned milk (i.e., evaporated milk, sweetened condensed milk), canned/bottled cheese (spreads, squirtable, grated Parmesan), freeze-dried cheese, cheese powder, frozen cheese, and ice cream. *Non-dairy beverages*: coffee (ground, beans, canned, bottled), black or green tea, herbal tea, canned/bottled/boxed juice, canned/bottled soda pop/soft drinks, powdered drink mix, alcohol, energy drinks, and sports drinks. *Other items*: jam/jelly/preserves, candy, baby formula/cereal/food, vitamin/mineral supplements, pet/animal food, and frozen meals/entrees.
Length of time food could last	
“About how many days, weeks or months could the food last to feed all members of your household based on normal portion sizes and meal patterns?”	*Definition*: “Think about all of the food you currently have in your household, including food in your refrigerator/freezer, food on your kitchen shelves, food stored for emergency situations, or any other food available in your home.” *Response options*: Less than 3 days, 3 days, more than 3 days but less than 1 week, at least 1 week but less than 1 month, at least 1 month but less than 3 months, at least 3 months but less than 6 months, at least 6 months but less than 1 year, and 1 year or more.
Water storage	
“Do you have water stored in containers that you could drink in an emergency situation?”	*Definition*: “This includes water stored in any package, of any container size or amount.” *Response options*: Yes, no
Type of water stored	
“Which type of water do you have stored that you could drink in an emergency situation?”	*Definition*: “’Commercially packaged water’ is prepackaged water purchased at a store. This water is often packaged in plastic bottles or jugs.” *Response options*: Mark all that apply. Containers filled with tap water, commercially packaged water
Length of time water could last	
“About how many days, weeks, or months could your stored water last for all members of your household if you allow for 1 gallon of water per person per day?”	*Definition*: “Think about all of the water you have stored in containers that could be used for drinking water in an emergency situation," and "Note that 1 gallon is the size of a milk jug, 8 water bottles (500 mL/16.9 fl oz), 2 soda bottles (2-L).” *Response options*: Less than 3 days; 3 days; more than 3 days, but less than 1 week; at least 1 week, but less than 1 month; at least 1 month, but less than 3 months; and more than 3 months.
Disaster supplies kit	
“Does your household have at least one “disaster supplies kit” that you could take with you if you had to leave your home on short notice?”	*Definition*: “A ‘disaster supplies kit’ (also known as a 72-h kit) is a collection of basic items that include food, water, and other emergency supplies, stored in a portable container, for use in the event of a disaster.” *Response options*: Yes, no
Accessible cash	
“Considering all of the accessible cash you have, how long do you think it would last to provide basic needs for your household?”	*Definition*: “Cash stored in your home, checking account, savings account, and any other account that you could access to provide basic needs (food, shelter, etc.) for your household if your regular household income was not available.” *Response options*: less than 1 week; at least 1 week, but less than 1 month; at least 1 month, but less than 3 months; at least 3 months, but less than 6 months; at least 6 months, but less than 9 months; at least 9 months, but less than 12 months; and at least 12 months.
Respondents’ perceived responsibility of household, local, state, and federal government	
“It is the federal government’s [state government’s/local government’s/an individual household’s] responsibility to take care of my household in a large-scale disaster or emergency.”	*Response options*: 5-point Likert scale (strongly disagree to strongly agree)

* For respondents who reported having at least one freezer in combination with a refrigerator and/or stand-alone upright or chest freezer. ^†^ Response option in frozen foods and non-perishable foods survey questions.

**Table 2:** Demographic characteristics of food assistance program participants and non-participants across the United States, using chi-square statistics, Fisher’s exact test, and independent *t*-tests (*n* = 572).

Respondent Characteristics	Total	Food Assistance Program Participants	Non-Participants	*p*-Value
		(*n* = 126) *	(*n* = 446)	
	*n (%)*			
Sex **				0.08
Male	196 (34.3)	35 (27.8)	161 (36.2)	
Female	375 (65.7)	91 (72.2)	284 (63.8)	
**Age (in years)**				0.0001
18 to 44	277 (48.4)	75 (59.5)	202 (45.3)	
45 to 64	180 (31.5)	42 (33.3)	138 (30.9)	
65 and older	115 (20.1)	9 (7.1)	106 (23.8)	
**Race/Ethnicity ^†^**				<0.008
Alaska Native	1 (0.2)	0 (0)	1 (0.2)	
American Indian	4 (0.7)	0 (0)	4 (0.9)	
Asian	15 (2.6)	3 (2.4)	12 (2.7)	
Black or African American	24 (4.2)	11 (8.7)	13 (2.9)	
Hispanic or Latino	13 (2.3)	7 (5.6)	6 (1.4)	
Pacific Islander	1 (0.17)	0 (0)	1 (0.2)	
White	484 (84.6)	96 (76.2)	388 (87.0)	
Other ^a^	7 (1.2)	3 (2.4)	4 (0.9)	
Multiracial/ethnic ^b^	23 (4.0)	6 (4.8)	17 (3.8)	
**Education Level**				<0.0001
Less than high school	14 (2.5)	7 (5.6)	7 (1.6)	
Received high school diploma or G.E.D.	96 (16.8)	32 (25.4)	64 (14.4)	
Some college or technical school	153 (26.8)	42 (33.3)	111 (24.9)	
Completed technical school or	95 (16.6)	23 (18.3)	72 (16.1)	
2-year degree/program				
Completed 4-year college or university degree	143 (25.0)	17 (13.5)	126 (28.3)	
Completed an advanced graduate/professional degree	71 (12.4)	5 (4.0)	66 (14.8)	
**Employment Status**				<0.0001
Full time	160 (28.0)	24 (19.1)	136 (30.5)	
Part time	64 (11.2)	18 (14.3)	46 (10.3)	
Unemployed	67 (11.7)	27 (21.4)	40 (9.0)	
Retired	139 (24.3)	16 (12.7)	123 (27.6)	
Other	142 (24.8)	41 (32.5)	101 (22.7)	
**Residence Type of Building**				0.0008
Stand-alone house	388 (67.8)	72 (57.1)	316 (70.9)	
Duplex/Townhouse	30 (5.2)	9 (7.1)	21 (4.7)	
Apartment/Condo	131 (22.9)	33 (26.2)	98 (22.0)	
Mobile home	23 (4.0)	12 (9.5)	11 (2.5)	
**U.S. Geographical Region ^‡^**				0.05
Midwest	58 (10.1)	19 (15.1)	39 (8.7)	
Northeast	54 (9.4)	12 (9.5)	42 (9.4)	
South	85 (15.9)	24 (19.1)	61 (13.7)	
West	375 (65.6)	71 (56.4)	304 (68.2)	
Rural-Urban Commuting Area ^†,^***				0.27
*Metropolitan area core:* primary flow within an urbanized area (UA)	466 (81.6)	96 (76.2)	370 (83.2)	
*Metropolitan area high commuting*: primary flow 30% or more to a UA	23 (4.0)	5 (4.0)	18 (4.0)	
*Micropolitan area core*: primary flow within an Urban Cluster (UC) of 10,000 to 49,999 (large UC)	44 (7.7)	16 (12.7)	28 (6.3)	
*Micropolitan high commuting*: primary flow 30% or more to a large UC	8 (1.4)	1 (0.8)	7 (1.6)	
*Micropolitan low commuting*: primary flow 10% to 30% to a large UC	2 (0.4)	0 (0)	2 (0.5)	
*Small town core*: primary flow within an Urban Cluster of 2500 to 9999 (small UC)	13 (2.3)	4 (3.2)	9 (2.0)	
*Small town high commuting*: primary flow 30% or more to a small UC	2 (0.4)	1 (0.8)	1 (0.2)	
Rural areas: primary flow to a tract outside a UA or UC	13 (2.3)	3 (2.4)	10 (2.3)	
**Food Assistance Program Usage ^††^**	126 (22.0)		--	--
SNAP	84 (14.7)	84 (66.7)	--	
WIC	26 (4.6)	26 (20.6)	--	
Free School Meals	24 (4.2)	24 (19.0)	--	
Reduced-price School Meals	8 (1.4)	8 (6.3)		
Food bank	30 (5.2)	30 (23.8)	--	
Religious organization	15 (2.6)	15 (11.2)	--	
**Length of Time Accessible Cash Could Provide Basic Needs to Household ^‡‡^**				<0.0001
Less than 1 week	137 (24.0)	50 (40.0)	87 (19.6)	
At least 1 week, but less than 1 month	118 (20.7)	35 (28.0)	83 (18.7)	
At least 1 month, but less than 3 months	122 (21.4)	24 (19.2)	98 (22.0)	
At least 3 months, but less than 6 months	70 (12.3)	11 (8.8)	59 (13.3)	
At least 6 months or more	123 (21.6)	5 (4.0)	118 (26.5)	
**Perceived Overall Health**				0.0002
Poor	16 (2.8)	4 (3.2)	12 (2.7)	
Fair	102 (17.8)	38 (30.2)	64 (14.4)	
Good	227 (39.7)	51 (40.5)	176 (39.5)	
Very Good	181 (31.6)	28 (22.2)	153 (34.3)	
Excellent	46 (8.0)	5 (4.0)	41 (9.2)	
	*mean ± SE*			
**Number of Adults in Household**	2.1 ± 0.04	2.1 ± 0.1	2.1 ± 0.04	0.8
**Number of Children (<18 years) in Household**	0.7 ± 0.05	1.2 ± 0.1	0.5 ± 0.1	<0.0001

* Food assistance program participants based on respondents’ report of using one or more of these programs: the Special, Supplemental Nutrition Program for Women, Infants and Children (WIC), the Supplemental Nutrition Assistance Program (SNAP), food bank, religious organization resources, reduced-price school breakfast/lunch, free school breakfast/lunch, or other food assistance program (as defined by respondent). ** Missing data, *n* = 1. ^†^ Fisher’s exact test used for comparison between Food Assistance Program Participants and Non-participants because of the high percentage of cells that had expected counts less than 5. **^‡^** Respondents’ state of residence in the United States (US) was classified into a geographical region based on the US Census Bureau classifications [15]: West = Alaska, Arizona, California, Hawaii, Idaho, Montana, Nevada, New Mexico, Oregon, Utah, and Washington; South = Alabama, Arkansas, Delaware, Florida, Georgia, Kentucky, Louisiana, Maryland, Mississippi, North Carolina, Oklahoma, Tennessee, Texas, Virginia, and West Virginia; Northeast = Connecticut, Maine, Massachusetts, New Hampshire, New Jersey, New York, Pennsylvania, and Rhode Island; and Midwest = Illinois, Indiana, Iowa, Kansas, Michigan, Minnesota, Missouri, Nebraska, North Dakota, Ohio, South Dakota, and Wisconsin. *** Respondents’ zip code was used to classify into Rural-Urban Community Area Codes as outlined by the US Department of Agriculture [16]. One respondent’s data was excluded because of inconsistencies among reported zip code, state, and county of residence. ^††^ Totals in the columns do not equal 126 because respondents could select all options that applied for this question, meaning some respondents indicated participating in more than one food assistance program. The 126 (22%) is the total number of participants who indicated using at least one food assistance program. **^‡‡^** Missing data, *n* = 2; accessible cash defined as cash stored in your home, checking account, savings account, and any other account that you could access to provide basic needs (food, shelter, etc.) for your household if your regular household income was not available. ^a^ Defined by respondents as Persian (*n* = 1), Afro-Latino (*n* = 1), Black (Caribbean, *n* = 1), Portuguese (*n* = 1), human (*n* = 1), French Creole/Portuguese (*n* = 1), and mixed-race (*n* = 1). ^b^ Defined by respondents as Black or African American and American Indian (*n* = 1); Black or African American and White (*n* = 2); American Indian, Asian, White, Hispanic, and Native Hawaiian (*n* = 1); American Indian and Hispanic (*n* = 2); American Indian and White (*n* = 6); Asian, Hispanic, and Pacific Islander (*n* = 1); Asian and White (*n* = 6); Hispanic and White (*n* = 3), and Other (disabled) and White (*n* = 1).

**Table 3:** Perceptions of emergency preparedness among food assistance program participants and non-participants across the United States, using chi-square statistics and independent *t*-tests (*n* = 572).

Response Item	Total	Food Assistance Program Participants (*n* = 126) *	Non-Participants (*n* = 446)	*p*-Value
	*n (%)*			
**Household Preparedness Level for Large-scale Disaster or Emergency ^†^**				
Overall Preparedness				0.20
Well prepared	51 (8.9)	14 (11.1)	37 (8.3)	
Somewhat prepared	307 (53.7)	59 (46.8)	248 (55.6)	
Not at all prepared	214 (37.4)	53 (42.1)	161 (36.1)	
Providing Water for Household				0.75
Well prepared	105 (18.4)	22 (17.5)	83 (18.6)	
Somewhat prepared	252 (44.1)	53 (42.1)	199 (44.6)	
Not at all prepared	215 (37.6)	51 (40.5)	164 (36.8)	
Providing Food for Household				0.98
Well prepared	144 (25.2)	31 (24.6)	113 (25.3)	
Somewhat prepared	312 (54.5)	69 (54.8)	243 (54.5)	
Not at all prepared	116 (20.3)	26 (20.6)	90 (20.2)	
**Own a Disaster Supplies Kit**				0.93
Yes	188 (32.9)	41 (32.5)	147 (33.0)	
No	384 (67.1)	85 (67.5)	299 (67.0)	
	*mean ± SE*			
Perceived Government and Individual Responsibility for Emergency Response and Preparedness ^‡,^**				
“It is the federal government’s responsibility to take care of my household in a large-scale disaster or emergency.”	2.7 ± 0.05	2.8 ± 0.1	2.6 ± 0.1	0.18
“It is the state government’s responsibility to take care of my household in a large-scale disaster or emergency.”	2.8 ± 0.05	2.9 ± 0.1	2.8 ± 0.1	0.32
“It is the local government’s responsibility to take care of my household in a large-scale disaster or emergency.”	2.9 ± 0.05	2.9 ± 0.1	2.8 ± 0.1	0.46
“It is an individual household’s responsibility to take care of their own household in a large-scale disaster or emergency.”	4.2 ± 0.03	4.1 ± 0.1	4.2 ± 0.04	0.16

* Food assistance program participants based on respondents’ report of using one or more of these programs: the Special, Supplemental Nutrition Program for Women, Infants and Children (WIC), the Supplemental Nutrition Assistance Program (SNAP), food bank, religious organization resources, reduced-price school breakfast/lunch, free school breakfast/lunch, or other food assistance program (as defined by respondent). ^†^ Large-scale disaster or emergency defined on the survey as “any event that leaves you isolated in your home or displaces you from your home for at least 3 days”. ^‡^ Response options were a 5-point Likert scale (1 = Strongly Disagree to 5 = Strongly Agree). ** Missing data, *n* = 1.

**Table 4:** Food storage practices for large-scale disasters or emergencies among food assistance program participants and non-participants across the United States, using chi-square statistics and independent *t*-tests (*n* = 572).

Response Item	Total	Food Assistance Program Participants (*n* = 126) *	Non-participants (*n* = 446)	*p*-Value
	*mean ± SE*			
**Number of Different Kinds Non-perishable and Frozen Foods Available at Home for Large-scale Disaster or Emergency**				
Grains and Cereal-based Foods ^1^	10.7 ± 0.16	10.5 ± 0.4	10.7 ± 0.2	0.60
Fruits ^2^	1.4 ± 0.05	1.4 ± 0.1	1.4 ± 0.1	0.82
Vegetables ^3^	2.8 ± 0.07	3.1 ± 0.1	2.8 ± 0.1	0.10
Protein Foods ^4^	4.3 ± 0.09	4.2 ± 0.2	4.3 ± 0.1	0.47
Dairy ^5^	2.2 ± 0.07	2.6 ± 0.2	2.1 ± 0.1	0.01
	*n (%)*			
**Other Foods Available at Home for Large-scale Disaster or Emergency**				
Non-perishable non-dairy beverages ^6^	549 (96.0)	120 (95.2)	429 (96.2)	0.63
Jam/jelly/preserves	454 (79.4)	104 (82.5)	350 (78.5)	0.32
Candy	354 (61.9)	77 (61.1)	277 (62.1)	0.84
Baby formula/cereal/food	64 (11.2)	25 (19.8)	39 (8.7)	0.0005
Vitamin/mineral supplements	417 (72.9)	76 (60.3)	341 (76.5)	0.0003
Pet/animal food	327 (57.2)	78 (61.9)	249 (55.8)	0.22
Frozen Meals/Entrees	156 (27.3)	41 (32.5)	115 (25.8)	0.13
**Length of Time Food Available in the Home Could Feed Household**				0.91
Less than 3 days	24 (4.2)	4 (3.2)	20 (4.5)	
3 days	21 (3.7)	5 (4.0)	16 (3.6)	
More than 3 days, but less than 1 month	268 (46.9)	58 (46.0)	210 (47.1)	
At least 1 month or more	259 (45.3)	59 (46.8)	200 (44.8)	

* Food assistance program participants based on respondents’ report of using one or more of these programs: the Special, Supplemental Nutrition Program for Women, Infants and Children (WIC), the Supplemental Nutrition Assistance Program (SNAP), food bank, religious organization resources, reduced-price school breakfast/lunch, free school breakfast/lunch, or other food assistance program (as defined by respondent). ^1^ *Grains and Cereal-based foods* calculated for each household based on the number of items respondents reported having from this list of 20 foods: brown rice, white rice, wheat kernels/unground wheat, oats/oatmeal, ready-to-eat cereal, pasta, packaged rice, or pasta seasoned meals/side dishes, cornmeal/masa flour, whole wheat flour, white flour, low-sodium crackers, regular crackers, pretzels, corn chips/tortilla chips, pancake/waffle mix, granola bars or fruit grain bars, popcorn, cake mixes, packaged cookies, and bread/rolls/bagels/tortillas. ^2^ *Fruits* calculated for each household based on the number of items respondents reported having from this list of 5 foods: commercially canned/bottled fruits (not jams/jellies), home-canned/bottled fruits (not jams/jellies), commercially dried/dehydrated fruits (i.e., raisins, fruit leather, dried apricots), home-dried/dehydrated fruits (i.e., raisins, fruit leather, dried apricots), and frozen fruit. ^3^ *Vegetables* calculated for each household based on the number of items respondents reported having from this list of 8 foods: commercially canned/bottled vegetables, home canned/bottled vegetables, commercially dried/dehydrated vegetables (i.e., dried carrots, tomatoes), home dried/dehydrated vegetables (i.e., dried carrots, tomatoes), potato chips, instant mashed potatoes, packaged potato meals/side-dishes (i.e., scalloped, hash browns, cheesy), and frozen vegetables. ^4^ *Protein Foods* calculated for each household based on the number of items respondents reported having from this list of 13 foods: commercially canned/bottled meat (includes tuna or other fish, chicken/turkey, beef, pork, etc.), home canned/bottled meat (includes poultry, beef, pork, fish, etc.), commercially dried/dehydrated meats (i.e., jerky, bacon bits, fish or seafood, meat substitutes), home dried/dehydrated meats (i.e., jerky, bacon bits, fish or seafood, meat substitutes), frozen meats (includes poultry, beef, pork, fish, meat substitutes, etc.), commercially canned bottled beans/legumes (not green beans), home canned bottled beans/legumes (not green beans), dry beans/legumes, frozen beans/legumes (not green beans), nuts, sunflower seeds or pumpkin seeds, peanut butter, and trail mix. ^5^ *Dairy* calculated for each household based on the number of items respondents reported having from this list of 11 foods: powdered milk, powdered milk alternatives (i.e., soy, almond, rice), hot cocoa mix, boxed (shelf-stable) fluid cow’s milk, boxed (shelf-stable) fluid milk alternatives (i.e., soy, almond, rice), canned milk (i.e., evaporated milk, sweetened condensed milk), canned/bottled cheese (spreads, squirtable, grated Parmesan), freeze-dried cheese, cheese powder, frozen cheese and ice cream. ^6^ *Non-perishable non-dairy beverages* calculated for each household based on the frequency of having one or more of these non-perishable non-dairy beverages available at home: coffee (ground, beans, canned, bottled), black or green tea, herbal tea, canned/bottled/boxed juice, canned/bottled soda pop/soft drinks, powdered drink mix, alcohol, energy drinks, and sports drinks.

**Table 5:** Water storage practices for large-scale disasters or emergencies among food assistance program participants and non-participants across the United States, using chi-square statistics and independent *t*-tests (*n* = 572).

	Total	Food Assistance Program Participants (*n* = 126) *	Non-Participants (*n* = 446)	*p*-Value
	*n (%)*			
**Water Storage Availability for Emergency Situations**				0.55
Yes	358 (62.6)	76 (60.3)	282 (63.2)	
No	214 (37.4)	50 (39.7)	164 (36.8)	
**Type of Water Stored ^†^**				
Commercially-packaged containers	274 (47.9)	49 (38.9)	225 (50.5)	0.02
Personally-filled containers	151 (26.4)	38 (30.2)	113 (25.3)	0.28
**Length of Time Water Available in the Home Could Provide the Household**				0.05
No water storage	214 (37.4)	50 (39.7)	164 (36.8)	
Less than 3 days	57 (10.0)	19 (15.1)	38 (8.5)	
3 days	42 (7.3)	4 (3.2)	38 (8.5)	
More than 3 days, but less than 1 month	176 (30.8)	33 (26.2)	143 (32.1)	
At least 1 month or more	83 (14.5)	20 (15.9)	63 (14.1)	

* Food assistance program participants based on respondents’ report of using one or more of these programs: the Special, Supplemental Nutrition Program for Women, Infants and Children (WIC), the Supplemental Nutrition Assistance Program (SNAP), food bank, religious organization resources, reduced-price school breakfast/lunch, free school breakfast/lunch, or other food assistance program (as defined by respondent). ^†^ Based on respondents who indicated having water storage available for emergency situations (*n* = 358). Respondents could mark all options that applied; thus, totals do not add up to 358.

## Data Availability

The data presented in this study are available upon request from the corresponding author. The data are not publicly available due to privacy.
